# Inflammatory Biomarkers for Outcome Prediction in Patients With Metastatic Testicular Cancer

**DOI:** 10.3389/fonc.2022.910087

**Published:** 2022-06-10

**Authors:** Sara Bleve, Maria Concetta Cursano, Chiara Casadei, Giuseppe Schepisi, Cecilia Menna, Milena Urbini, Caterina Gianni, Silvia De Padova, Alessia Filograna, Valentina Gallà, Giovanni Rosti, Domenico Barone, Michal Chovanec, Michal Mego, Ugo De Giorgi

**Affiliations:** ^1^ Department of Medical Oncology, IRCCS Istituto Romagnolo per lo Studio dei Tumori (IRST) “Dino Amadori”, Meldola, Italy; ^2^ Biosciences Laboratory, IRCCS Istituto Romagnolo per lo Studio dei Tumori (IRST) “Dino Amadori”, Meldola, Italy; ^3^ Psycho-Oncology Unit, IRCCS Istituto Romagnolo per lo Studio dei Tumori (IRST) “Dino Amadori”, Meldola, Italy; ^4^ Unit of Biostatistics and Clinical Trials, IRCCS Istituto Romagnolo per lo Studio dei Tumori (IRST) “Dino Amadori”, Meldola, Italy; ^5^ Radiology Unit, IRCCS Istituto Romagnolo per lo Studio dei Tumori (IRST) “Dino Amadori”, Meldola, Italy; ^6^ 2nd Department of Oncology, Comenius University, Faculty of Medicine, National Cancer Institute, Bratislava, Slovakia

**Keywords:** germ cell tumors, inflammation markers, immunity, chemotherapy, testicular cancer (GCT), prognostic factors

## Abstract

Germ cell tumors are the most common malignant tumors in male young adults. Platinum-based chemotherapy has dramatically improved the outcome of metastatic germ cell tumor patients and overall cure rates now exceed 80%. The choice of medical treatment can be guided by the prognosis estimation which is an important step during the decision-making process. IGCCCG classification plays a pivotal role in the management of advanced disease. However, histological and clinical parameters are the available factors that condition the prognosis, but they do not reflect the tumor’s molecular and pathological features and do not predict who will respond to chemotherapy. After first-line chemotherapy 20%-30% of patients relapse and for these patients, the issue of prognostic factors is far more complex. Validated biomarkers and a molecular selection of patients that reflect the pathogenesis are highly needed. The association between cancer-related systemic inflammation, tumorigenesis, and cancer progression has been demonstrated. In the last years, several studies have shown the prognostic utility of immune-inflammation indexes in different tumor types. This review analyzed the prognostic impact of inflammatory markers retrieved from routine blood draws in GCT patients.

## Introduction

Germ cell tumors (GCTs) are the most common malignant tumors in male adults aged 15-40 years and represent the major cause of death attributable to cancer in this population (1–3). Platinum-based chemotherapy has dramatically improved the outcome of these patients, especially those with advanced seminoma disease, with overall cure rates that now exceed 80% (4). Non-seminoma histotype is associated with poorer outcomes and lower sensitivity to chemotherapy and radiotherapy (5).

Prognosis and choice of treatment are correlated with various clinical and histological features. After first-line chemotherapy 20%-30% of patients relapse and for these patients, the identification of prognostic factors is more complex (6). Validated biomarkers and a molecular selection of patients that reflect the pathogenesis are highly needed to better address treatment and improve survival rates. As it has been demonstrated, the tumor microenvironment and the associated host inflammatory response have an important role in proliferation, survival of malignant cells, angiogenesis, metastasis, and a reduced response to chemotherapeutic agents (7, 8).

Moreover, recently, several studies have increased the interest in several immune-inflammation indexes, showing their prognostic utility in several tumors (9).

In this paper, we review the current knowledge on the relationship between inflammation and cancer, focusing on the possible prognostic and predictive role of inflammation markers in patients affected by testicular GCTs.

## Treatment Options and Prognostic Factors

An overall long-term disease-free status can be achieved in the majority of metastatic GCT patients with multimodality therapy including cisplatin-based combination chemotherapy and subsequent resection of residual disease (5). The standard treatment of metastatic disease is represented by 3-4 cycles of bleomycin/etoposide/cisplatin (10, 11). Using ifosfamide instead of bleomycin can be considered in selected cases with intermediate/poor prognosis since it has the same efficacy but ifosfamide is associated with more toxicity and a major rate of sterility (12, 13).

The international Germ-Cell Cancer Cooperative Group (IGCCCG) score is a guide for treatment decisions in the daily routine for metastatic patients. It classifies patients with seminoma disease into two prognostic groups (good and intermediate risk) and patients with non-seminoma disease into three prognostic groups (good, intermediate, and poor risk), according to several clinical parameters (primary tumor site, metastatic sites, degree of serum tumor markers levels) (14).

Recently, a refinement of IGCCCG has been published demonstrating an improvement in the IGCCCG intermediate and poor-risk group’s survival probably due to the best supportive care and an optimal surgical approach to residual disease (15, 16). The centralization of patients in high-volume centers is essential for adequate clinical management, particularly in rare and life-threatening conditions like “choriocarcinoma syndrome” (17). In the recent IGCCCG-update, LDH with a cutoff of 2.5 upper normal limits was identified as an adverse prognostic factor in patients with good prognosis seminoma, which may suggest an intensification of treatment in this group. Advanced age and lung metastasis are included in the new IGCCG update model as additional adverse prognostic factors (15, 16). To help clinicians in the treatment choice, a web-based calculator was created on the evidence reported in the new IGCCCG update.

In patients with cisplatin-refractory testicular cancer, conventional-dose chemotherapy (CDCT) can induce an objective response in 10%-20% of patients. The most used regimens include VIP (cisplatin, etoposide, ifosfamide), VeIP (vinblastine, ifosfamide, cisplatin), and TIP (paclitaxel, ifosfamide, cisplatin) (18, 19). CDCT can induce long-term remissions in 30%-50% as second-line therapy in all patients, but it maintains efficacy in a small group of multiple-relapsed or cisplatin-refractory GCTs (20).

In the last years, the use of high-dose chemotherapy (HDCT) in hematologic and solid tumors has been spreading due to the great results in patient outcomes (21). HDCT use with the support of peripheral blood progenitor cells (PBPC) has been extensively studied in cisplatin-refractory GCT patients, and it has been demonstrated that it can induce durable remissions in a higher percentage of cases, mainly in those with unfavorable characteristics. The use of HDCT is a particularly valid option both in adult and pediatric patients with extra-gonadal GCT and brain metastases, which are notoriously associated with an inferior survival rate with CDCT (4, 22–24).

In GCT patients, HDCT has a therapeutic role either as a first-line or salvage setting, and its use in clinical practice is supported by the results of two large retrospective series and a prospective clinical trial (20, 25, 26). The combination of carboplatin and etoposide (two or three cycles rapidly repeated) is the standard regimen for HDCT (26). Mobilizing, withdrawing, and re-infusing a sufficient number of PBPC is the necessary condition to perform HDCT. Granulocyte-colony-stimulating factor (G-CSF) and chemotherapy are usually used to induce PBPC mobilization along with common drugs and schedules such as cisplatin, ifosfamide, paclitaxel (TIP), cisplatin, etoposide, and ifosfamide (PEI) and cisplatin and ifosfamide (PI) (27).

Since both CDCT and HDCT have shown curative potential in the management of relapsed/refractory GCTs, great efforts have been made to define prognostic factors that are able to orient clinical decisions. Nevertheless, there are no useful biomarkers to guide treatment selection in daily routines and it represents one of the major challenges in the clinical approach to GCT patients.

Lorch et al. developed the IPFSG, a prognostic model for patients who failed cisplatin-based first-line chemotherapy. This score is based on six variables (primary site, first-line response, platinum-free interval, presence of bone, liver or brain metastasis, tumor markers: human chorionic gonadotropin, β-HCG, and alpha-fetoprotein, AFP level at baseline of salvage chemotherapy) and it can divide patients into five prognostic groups: very low, low, intermediate, high, and very high risk (22). An IPFSG prognostic score may help clinical decisions; however, due to the high complexity of this population as well as the high treatment-related toxicity rates, the search for additional prognostic factors is necessary.

As current guidelines recommend, the treatment monitoring of these patients is largely based on the measurement of the classical serum tumor markers despite their low sensitivity and the different expression in the various subtypes (28). Only 50% of all GCTs express one of the three markers: seminoma does not express AFP and teratoma, the most differentiated subtype, lacks all markers expression and an informative biomarker of this subtype is still missing (29).

New prognostic and predictive factors are under development such as the serum levels of MicroRNA-371a-3p (M371 Test) that seem to outperform the classical markers in all clinical stages with a sensitivity and a specificity greater than 90% in a recent prospective study. This novel biomarker is a small non-coding RNA that regulates gene expression and it seems to be informative in both seminoma and non-seminoma subgroups except teratoma (30, 31).

Lobo et al. proposed the model of “microRNA switch” according to in differentiated teratoma microRNA-371a-3p is replaced by miR-885-5p which is a p53 activator, possibly contributing to the well-known cisplatin resistance of this subtype (32).

The strong evidence of the usefulness of this marker for GTC diagnosis and monitoring treatments needs further validation.

Susceptibility to apoptosis plays a pivotal role in the intrinsic sensitivity of tumor therapies.

P53 is a tumor suppressor and it causes the arrest of the cell cycle, induces apoptosis, and promotes cell repair or senescence in the presence of cell or DNA damage or abnormal cell growth conditions (33, 34).

Its functional inactivation is common in somatic cancer and it promotes tumorigeneses and leads to resistance to anti-cancer treatments (35, 36).

In TGCTs there is frequent overexpression of wild-type p53 and mutations are hardly found and this might explain the high treatment sensitivity of these tumors. P53 mutations may emerge in cisplatin-resistance tumors but their role is still debatable (35).

MDM2 is a ubiquitin ligase, and its principal function is the downregulation of p53 activity in forming a negative feedback loop with p53. Several reports have shown an overexpression of MDM2 in cisplatin-resistance tumors (37, 38).

Current knowledge suggests a contribution of the up/downregulation of MDM2/p53 to define the cisplatin resistance phenotype. It could have a clinical impact on the selection of patients eligible for high-dose chemotherapy that is based on a high dose of cisplatin, even if not all studies have shown the utility of this axis to predict disease recurrence (39).

It is well known that the chemosensitivity of specific tumors depends on their DNA damage and the activation of the DNA-damage response (DDR) Another reason for the high chemosensitivity of testicular cancer is its low expression levels of factors involved in the DNA repair system (32).

PARP Poly (ADP-ribose) polymerase (PARP) is a nuclear enzyme that repairs DNA single-strand breaks and PARP inhibitors represent an effective treatment in different malignancies. In the view of Homologous Recombination deficiency, there is a biological rationale for the use of PARP inhibitors in testicular cancer, and different trials that evaluate the clinical utility of this class of drugs are ongoing (39).

The importance of the recent results of immunotherapy in different tumors, and the efficacy of immune checkpoint inhibitors (ICIs) in testicular cancer, as reported in the literature by several case reports and small series, has drawn attention toits use for GTCs.

Several ongoing trials are evaluating immunotherapy in refractory testicular GCTs who relapse after HDCT with contradictory primary results (40–46). The lack of encouraging results in this setting is probably due to the immune tolerance of GCTs, and low tumor mutation burden with a low number of neo-antigens. Nevertheless, ICIs (both PDL-1 and CTL-4 inhibitors) have shown benefits in some patient groups, for example, in those affected by choriocarcinoma of the testis, ICIs are effective and expression of PD-L1 predicts response to treatment (47).

Other mechanisms to improve the outcome are related to the new delivery modality (48).

## Cancer-Related Inflammation and Its Prognostic Role

The first correlation between cancer and inflammation was discovered in the 19th century by Virchow and ever since the importance of the role of inflammation in mediating tumorigenesis and the progression and metastasis of cancer has been confirmed (7, 49, 50). About 15% of all malignancies are caused by infectious agents that create a status of chronic inflammation. It has been found that there’s a strong association between an increased risk of malignancy and chronic inflammation caused by chemical and physical agents, autoimmune diseases, and inflammatory conditions of unknown etiology. To support this association, epidemiological data have shown a decrease in tumor progression and mortality risk in patients treated with nonsteroidal anti-inflammatory drugs (NSAIDs) (51). The hallmark of cancer-related inflammation is represented by the presence of inflammatory cells and inflammatory mediators. Carcinogenesis is promoted by systemic inflammation that damages the immune response allowing tumor cells to escape from immune surveillance. “Immune editing” is a dynamic and complex process initiated by tumor cells in response to immune surveillance that leads to tumor progression and it is made up of three phases: elimination, equilibrium, and escape. Several biological processes such as the inhibition of apoptosis, promotion of genomic instability, angiogenesis, and metastatic spread mediate the complex role of inflammatory cells and altered immunity in aiding tumor immune escape (7, 49, 52). Peripheral blood cells such as leukocytes, including neutrophils, lymphocytes, monocytes, and platelets have an important role in the previously mentioned processes. Tumor proliferation and metastatic spread are promoted by neutrophils that are recruited by cancer-related chemokines and cytokines (e.g., IL-6 and TNF) which are highly present in advanced cancer patients and which are also associated with drug resistance (53, 54). Tumor-associated macrophages (TAMs), derived from monocytes, represent the major component of tumor infiltrate and they have a pivotal role in promoting tumor proliferation, invasion, and metastatic spread (7, 55). On the contrary, the host immune response is promoted by lymphocytes that induce apoptosis and inhibit cell proliferation (56). In systemic inflammation, low levels of CD4+T cells are often observed, resulting in less-effective immune surveillance. The association of inflammatory cytokines and chemokines produced by tumor cells and tumor-associated blood cells and malignant progression have been described in several types of cancer like IL-1b/IL-6 networks that were investigated in different preclinical models and have been found to be highly expressed in human colorectal and gastric cancer, reinforcing its possible role in mediating tumorigenesis. Many pro-inflammatory cytokines are involved in germ cell proliferation and exercise an effect on spermatogenetic cell differentiation, playing a role also in testicular cancer pathogenesis, promoting metastatic processes, migration, and neo-angiogenesis (57).

Given the link between inflammation and tumor biology, there is a biological explanation for the use of inflammatory markers to predict cancer outcomes. The prognostic role of immune-inflammatory cells has been proven in several types of cancer and their utility varies between different tumors.

The chronic inflammatory process and the high burden of disease cause a great presence of inflammatory markers in advanced tumors (58). Inflammation-based markers can easily be detected from routine blood tests and, for this reason, might be useful prognosticators. Single markers such as leukocytes, platelets, and hemoglobin, and non-circulating blood cell markers (albumin, PCR), have been shown to have prognostic information. Also, several indexes that combine single inflammation parameters have been demonstrated to improve the prediction of oncologic outcomes that can help clinicians in the management of patients with advanced disease (9, 59–61).

In the last years, several studies have shown that inflammatory markers retrieved from routine blood sands can be useful also in outcome prediction of patients with testicular cancer showing that systemic inflammation could influence response to treatment.

**Table 1** reports the associations of blood-based systemic inflammatory markers with prognosis and outcome of patients with metastatic GCTs.

**Table 1:** Overview of studies investigating the prognostic role of inflammatory biomarkers in metastatic testicular cancer.

Biomarker	Author (reference)	Type of study	No. of patients	Cut off	OS		Cut off	PFS	
					HR (95% CI)	P value		HR (95% CI)	P value
**Leucocytes-related markers**									
**ANC**	(62)	Retrospective	164	ANC>8000n/uL	12.9 (10.4-15.2)	0.006	–	–	–
**NRL**	(63)	Retrospective	146	NLR > 4.5	84.5 (2.2–3193.4)	0.017	–	–	–
	(62)	Retrospective	164	NLR>4	14.1 (12.3-16.0)	0.035	–	–	–
	(64)	Retrospective	690	NRL>3	3 (1.79-5.01)	<0.01	NRL >2	1.99 (1.27–3.12)	< 0.01
	(65)	Retrospective	62	NRL> 3.3	4.49 (1.86–0.82)	0.0008	NRL>3.3	3.68 (1.66–8.19)	0.001
**Platelets-** **Related markers**									
**SII**	(66)	Retrospective	171	SII<1003	0.16 (0.08–0.32)	<0.001	SII<1003	0.22 (0.12–0.41)	<0.001
	(63)	Retrospective	146	SII> 1428	12.5 (1.17–26.26)	0.037	–	–	–
	(65)	Retrospective	62	SII≥844	3.00 (1.34-6.69)	0.007	SII>844	3.02 (1.47–6.20)	0.003
**CRP and Albumin-related markers**									
**CRP**	(63)	Retrospective	146	Higher PCR	6.44 (2.04–20.29)	0.001	–	–	–
**Albumin**	(63)	Retrospective	146	Lower albumin	0.844 (0.742–0.96)	0.010	–	–	–
**GPS**	(67)	Retrospective	63	GPS 0-1	9.97 (1.16–85.8)	0.04	GPS 0-1	4.56 (1.52–3.73)	<0.01
**PDL-1 on tumor cells**									
	(68)	Retrospective	140	QS<10	0.43 (0.15–1.23)	0.0397	QS<10	0.40 (0.16–1.01)	0.0081
**PDL-1 on TILs**									
	(69)	Retrospective	240	HS ≥ 160	0.08 (0.04 – 0.16)	0.001	HS ≥ 160	0.17 (0.09 – 0.31)	0.0006

ANC, absolute neutrophil count; NRL, neutrophil-lymphocyte ratio; SII, systemic inflammatory index; CRP, C-reactive protein; GPS, Glasgow prognostic score; HR, hazard ratio; CI, confidence interval; OS, overall survival; PFS, progression free survival.

## Leucocytes and Platelets-Related Markers

In cancer-associated systemic inflammatory response, the circulating neutrophil count is often increased and it is known that it plays a role in cancer progression. This pro-inflammatory status leads to lymphocytes suppression and activates T cells with a reduction of cytotoxic immune response to cancer. A high count of neutrophils and a low count of lymphocytes, also involved in primary and acquired drug resistance, have a prognostic value in several malignancies, but regarding testicular cancer, there is a limited number of studies that have investigated the value of the absolute count of neutrophils and lymphocytes (56, 70, 71).

Herraiz-Raya et al. in a retrospective cohort study evaluated different markers collected by blood sampling and their effect on patient prognosis. One of these was the absolute count of neutrophils and it has been shown that a value > 8000/μL was associated with higher percentages of progression and exitus (62).

The most studied index is the neutrophil-to-lymphocyte ratio (NLR) in several types of cancer, even if the mechanism of its strong prognostic value is not fully understood. NLR is defined as the absolute neutrophils count divided by absolute lymphocytes count and it is an inexpensive marker that can be easily acquired from the single complete blood cell parameters.

An increased NRL is associated with poor outcome and its prognostic value varies according to the type of cancer, disease stage, and treatment received, and the cut-off established is different in each study.

A meta-analysis of 100 studies involving 40,000 patients with several types of cancer has shown that an NLR of 4 was associated with an adverse overall survival (58).

High NRL was also demonstrated to be a prognosticator in urinary system cancer in a meta-analysis led by Wang (72).

Moreover, several studies focused on NLR in GCTs. Most of these investigated its role in the pre-orchiectomy setting where a high NLR is associated with advanced cancer stage and poor prognosis. The first study was carried on by Bolat et al. and concluded that NRL is not a biomarker of prognosis in testicular cancer but the study showed some limits such as the small sample size considered and a poor ROC value of the NRL cut off (73). A possible role of this parameter to predict the prognosis was suggested by Jankovich et al. in a study in which an NRL < 4.0 has been found in non-metastatic testicular cancer (74). Particularly NLR appears to be a useful marker for predicting localized and non-localized testicular GCT in the early postoperative period.

A significant decrease in NLR after orchiectomy, especially a value of less than 2, indicated localized disease in the study of Ilktac et al. On the contrary, the absence of a significant reduction after orchiectomy, in particular, an NLR value greater than 2, indicated non-localized disease (75).

In a recent retrospective study, preoperative NRL has been evaluated in 152 patients undergoing radical orchiectomy divided into good, moderate, and poor prognosis groups compared to a control group of healthy patients of similar age showing a significant difference in NLR between enrolled patients and control group (the cut off for NRL was found as 2.39) (76). Furthermore, the NRL value was significantly higher in patients with intermediate and poor prognosis than in patients with good prognosis (NLR cut-off value of 2.5) (76). Similarly, Tan reviewed the largest cohort of patients (160) with a diagnosis of testicular GCT on long-term postoperative follow-up and he demonstrated that NRL can be a useful marker to predict advanced GCT staging and poor survival outcomes. An NLR >3.00 was associated with advanced disease and poor cancer-specific survival, suggesting that adding the NLR value to the traditional cancer stage could help in identifying a group of high-risk patients who may benefit from closer surveillance or adjuvant therapy (77).

Fankhauser et al. demonstrated that different systemic inflammatory markers, including NRL, offer prognostic information in addition to IGCCCG risk groups for patients with advanced disease undergoing first-line chemotherapy. He identified that a high neutrophil count and high NRL were independent prognostic factors beyond the IGCCCG risk and that they were associated with a worse prognosis (63).

Similar results have been obtained in the recent retrospective study of Ribnikar who investigated the prognostic utility of NRL in a metastatic setting. He found that NRL >2 and >3 were associated with a worse PFS and OS, respectively, in patients with metastatic testicular cancer receiving first-line chemotherapy. Nevertheless, the multivariable analysis, including inflammatory markers and IGCCCG risk classification, revealed that NLR is not an independent predictor of PSF and OS (64).

A single retrospective trial evaluated the prognostic role of inflammatory indexes in relapsed/refractory germ cell tumors studying the correlation between baseline NRL, platelet-lymphocytes ratio (PLR), and systemic immune-inflammation index (SII) and response to HDCT, OS, and PFS in 62 patients undergoing HDCT. In this setting, NRL was a prognostic independent factor for OS and PFS (65).

Cytokines and chemokines produced by platelets play a key role in cancer-associated inflammation. They promote tumorigenesis by facilitating angiogenesis, tumor growth, and invasion. Several studies have shown that serum platelet levels are a prognostic factor in different solid tumors (9, 54, 78, 79).

Herraiz-Raya et al. analyzed the prognostic role of the absolute count of platelets and the platelet-lymphocyte ratio. Patients with an absolute count of platelets >400,000 and with a value of PLR >150 had a higher percentage of residual disease and a progression to stage II and III after the diagnosis (62).

To provide prognostic information in patients with malignant tumors, a prognostic indicator based on counts of neutrophils, lymphocytes, and platelets, called systemic immune inflammation index (SII), has been recently developed. It is calculated using the following formula: SII=PxN/L (platelets*neutrophils/lymphocytes) and it has been demonstrated to be more useful to predict oncologic outcomes compared to the ones based on single markers. High SII is linked to progression, metastasis, and poor outcome in several types of cancer (78, 80–82).

In a recent retrospective study, Lolli et al. identified SII as a strong parameter of prognostic and predictive outcomes in RCC patients treated with sunitinib (9).

In the first translational study, Chovanec et al. had shown the prognostic value of SII and its association with outcomes in patients with GCTs (66). The authors found that SII calculated before chemotherapy was an important indicator of prognosis. Indeed, a high SII was associated with shorter PFS and OS. Moreover, by combining SII with PD-L1 expression on tumor-infiltrating lymphocytes three distinctive prognostic groups were identified: the best prognosis was seen in patients with high expression of PD-L1 on TILs and a low SII, while the worst prognosis was seen in patients with a low expression of PDL1 and a high SII. Patients with SII and PD-L1 on TILs both low or high had an intermediate prognosis (66).

The prognostic utility of SII in patients undergoing first-line chemotherapy for GCT was confirmed by Fankhauser et al. showing that a high SII was an independent predictor of OS besides the IGCCCG risk groups and it was associated with a worse prognosis (63).

Finally, in the retrospective study led by Cursano, SII was an independent predictor of PSF and OS in patients with relapsed GCTs treated with salvage HDCT (65).

### CRP and Albumin-Related Markers

Other than an inflammatory status with the production of cytokines activating phase acute, the tumor is characterized by a reduction of noble proteins (albumin, pre-albumin, transferrin). Hypoalbuminemia is associated with a negative prognosis and reflects cancer-induced malnutrition. Cytokines derived from different inflammatory stimuli can be defined as a marker for systemic inflammation and they can be an important predictor of survival in several cancers including urological cancer (83–85). CRP is an acute-phase protein produced in hepatocytes, and a high CRP level is associated with poor outcomes in patients with cancer. CRP can be considered an important marker because it is inexpensive and easy to evaluate, even if it lacks a cut-off in each study (85). Despite few data demonstrating that CRP is associated with staging or survival in testicular cancer it was shown to be a biomarker of post-treatment complications in testicular cancer survivors.

A retrospective study of 539 testicular survivals showed that patients with CRP ≥ 1.5 mg/L had a larger risk of developing a non-germ cell second tumor and a higher risk of cardiovascular disease than survivors with CRP<1.5 mg/L. This suggests that CRP may serve as a potential marker of cardiovascular events in long-survival testicular cancers (86).

In GCTs the only study that evaluated the prognostic role of preoperative albumin and CRP level was led by Fankhauser which showed that they were useful in predicting RPLN involvement, distant metastasis, and prognosis. High PCR and low albumin levels were associated with lower OS at univariate analysis (63).

Glasgow prognostic score (GPS) is a score that includes the serum level of CRP and albumin reflecting the immunological and nutritional status of cancer patients. A high GPS seems to indicate an immune system imbalance that compromises the effective host-tumor immune response.

In several tumors, it has been demonstrated that GPS is a sensitive marker to predict the prognosis of patients with metastatic disease (87, 88).

Yoshinaga et al. evaluated different inflammation-based prognostic scores in patients with germ cell tumors and, studying the prognostic value of all of these, GPS has been demonstrated to be the most suitable biomarker of OS and PFS in patients with GCTs. Patients with a lower GPS had a significantly better OS and PFS than those with a higher GPS. It also considered PNI, a prognostic score based on two independent factors: serum albumin level and total lymphocyte count. The univariate analyses showed that PNI was associated with OS and PFS (67).

Cut-off values of these markers were lower than those used for other diseases due to the more favorable prognosis of GCTs compared to the other cancers and higher basal levels of albumin in these patients.

### PD-1/PD-L1

PD-1 is a T-cell regulator and it is expressed in different circulating cells (CD4+ T cells, CD8+ T cells, natural killer cells, B cells, and monocytes) (89). Its ligand, PD-L1, promotes self-tolerance by suppressing T cell inflammatory activity and it is an important mechanism by which cancer cells suppress the antitumor immune response (90). Indeed, alterations in the PD-1 pathway cause a great modification of immunological homeostasis. Many tumor cells express PD-L1 and PD-L2 with the inhibition of cytotoxic cells (91). In several cases, including advanced urothelial and genitourinary cancers, PD-L1 has become the strongest biomarker to predict the response to checkpoint inhibitors, a class of immunotherapy that blocks inhibitory signals mainly through the inhibition of the binding between PD-1 and PD-L1 or PD-L2 (92–97). In different studies, PD-L1 expression has been evaluated by immunohistochemistry (IHC) or IHC-based combined positive score (CPS) which is obtained as follows: CPS = 100 × PD-L1 stained cells (tumor cells, lymphocytes, macrophages)/total viable tumor cells.

In germ-cell tumors, PD-L1 expression has been observed in 73% and 64% of patients with seminoma and non-seminoma types, respectively, and its highest expression has been found in choriocarcinoma (98, 99).

In recent studies, a prognostic significance of PD-L1has been demonstrated: high levels in primary tumor tissue are associated with worse prognosis and poor-risk characteristics defined by International Germ Cell Cancer Collaborative Group (68). Another study investigated the prognostic role of PD-1 and PDL-1 expression on tumor-infiltrating lymphocytes showing that patients with high expression of PD-L1 had a better outcome (69). Chovanec et al. in their above-mentioned work recognized three different prognostic groups based on the systemic immune-inflammation index (SII) and PD-L1 expression on TILs. The prognosis was possibly mediated by PD-L1 expression on TILs that could reduce the cancer-related pro-inflammatory environment (66). The link between the immune infiltrates in terms of immune checkpoint PD-L1/CTLA-4 expression and the patient prognosis was studied by Lobo et al. in 162 samples. Infiltrating immune cells of all histological subtypes expressed PD-L1 and CTLA-4 while in tumor cells CTLA-4 expression was higher in yolk sac tumor, choriocarcinoma, and teratoma samples (99). The expression of PD-L1 and CTLA-4 in ICs was an independent predictor of better relapse-free survival (RFS) when adjusting for several other clinical variables (99). These results were confirmed by the study by Sadigh et al. in which it has been reported that PD-L1 wasn’t expressed on tumor cells of several histological samples except for choriocarcinoma while the other subtypes primarily expressed different levels of PD-L1 on tumor-associated macrophages (TAMs). Moreover, in this study it has been revealed that there is significantly higher expression of PD-L1 on TAMs in seminomatous compared to non-seminomatous samples (100).

The importance of the PD-1/PD-L1 signaling pathway in immune escape of testicular cancer was also studied by Siska who showed that increased PD-L1 expression and elevated PD-1/PD-L1 spatial interaction were predominantly found in seminomas and correlated with a good prognosis of the disease (101).

## Conclusion

The available prognostic scores for advanced testicular cancer help clinicians in deciding on treatment and are useful to determine the intensity of systemic first-line chemotherapy.

However, despite the great effectiveness of the treatments and the utility of these risk predictors, a subset of patients still relapse. The rarity of this pathology and the high complexity of this population as well as the high treatment-related toxicity requires the use of validated biomarkers that reflect the pathogenesis and biological features. Improved prediction of oncology outcomes might impact therapeutic decisions with the personalization of therapies and a better outcome for these patients.

It has been shown that host immune-inflammatory response to the tumor microenvironment is crucial in tumorigenesis and cancer progression and recent studies have demonstrated the utility of different inflammatory biomarkers to predict prognosis in cancer patients including those affected by testicular cancer. Systemic inflammatory markers that are easily retrieved from blood tests are very attractive and they seem to increment prognostic information in addition to the most used prognostic scores.

A higher number of prospective studies will be required to confirm their value and establish an optimal cutoff.

## Author Contributions

SB, MCC, GR, DB, and UD conceived and designed the article. SB and MCC conceived and created tables. SB, GS, CM, MU, MM, CC, AF, VG, and SD contributed to the writing of the manuscript. All authors contributed to the article and approved the submitted version.

## Funding

This work was partly supported thanks to the contribution of Ricerca Corrente by the Italian Ministry of Health within the research line: Precision, gender and ethnicity-based medicine and geroscience: genetic-molecular mechanism in the development, characterization and treatment of tumors.

## Conflict of Interest

UD received speaker honoraria or travel support from Astellas, Janssen-Cilag, and Sanofi-Aventis.

The remaining authors declare that the research was conducted in the absence of any commercial or financial relationships that could be construed as a potential conflict of interest.

## Publisher’s Note

All claims expressed in this article are solely those of the authors and do not necessarily represent those of their affiliated organizations, or those of the publisher, the editors and the reviewers. Any product that may be evaluated in this article, or claim that may be made by its manufacturer, is not guaranteed or endorsed by the publisher.
